# The Blue Problem: OLED Stability and Degradation Mechanisms

**DOI:** 10.1021/acs.jpclett.3c03317

**Published:** 2024-01-23

**Authors:** Eglė Tankelevičiūtė, Ifor D. W. Samuel, Eli Zysman-Colman

**Affiliations:** † Organic Semiconductor Centre, EaStCHEM School of Chemistry, University of St Andrews, St Andrews, U.K., KY16 9ST; ‡ Organic Semiconductor Centre, School of Physics & Astronomy, University of St Andrews, St Andrews, U.K., KY16 9SS

## Abstract

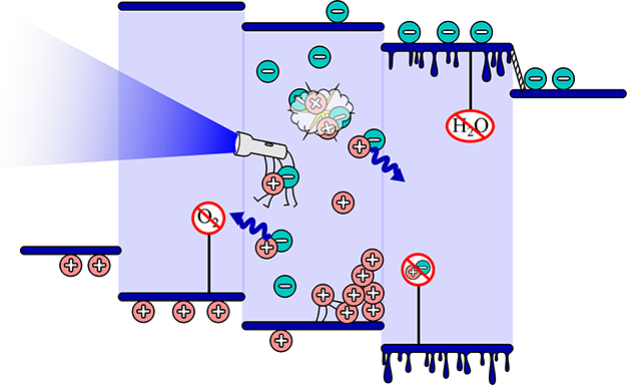

OLED technology has revolutionized the display industry and is promising for lighting. Despite its maturity, there remain outstanding device and materials challenges to address. Particularly, achieving stable and highly efficient blue OLEDs is still proving to be difficult; the vast array of degradation mechanisms at play, coupled with the precise balance of device parameters needed for blue high-performance OLEDs, creates a unique set of challenges in the quest for a suitably stable yet high-performance device. Here, we discuss recent progress in the understanding of device degradation pathways and provide an overview of possible strategies to increase device lifetimes without a significant efficiency trade-off. Only careful consideration of all variables that go into OLED development, from the choice of materials to a deep understanding of which degradation mechanisms need to be suppressed for the particular structure, can lead to a meaningful positive change toward commercializable blue devices.

The advent of organic semiconductors opened a new era of electronic consumer products, giving rise to robust and lightweight devices that can be easily tailored by changing the chemical structure of their constituent materials.^1^ Conjugated organic molecules can be used to make a range of devices such as light-emitting diodes (LEDs), solar cells, field-effect transistors, and lasers. In particular, the adoption of organic LEDs (OLEDs) has revolutionized the display industry, owing to their high efficiency, lightweight, fast response time, and superb image quality. Furthermore, OLEDs can be fabricated to be flexible or transparent, leading to new products in mobile phone, smartwatch, and TV markets. Despite their wide adoption and commercial success, the main outstanding issue for these devices is their lifetime, which is limited by the stability of the blue pixel. The suboptimal performance of blue OLEDs affects not only the stability of the device but also its energy efficiency. The high photon energy required for the materials to achieve deep-blue emission is also their Achilles heel, as this is also the origin of their photochemical instability and the rapid degradation of the device. To address this known issue of device stability requires methodical research into both the design of the materials within the device and the optimization of the device structure.^2^

An OLED consists of several layers of thin films of organic materials (on the order of tens of nanometers in thickness) sandwiched between two electrodes. When voltage is applied, electrons and holes are injected from the cathode and anode, respectively, and drift via charge transport layers to the emission layer (EML) where they recombine to form an exciton, which, in turn, decays radiatively to produce light. For charge transport layers, the choice of materials largely depends on their charge transport properties (i.e., charge mobilities) and energy levels, whereas those within the EML must be tuned to not only maintain optimal charge transport to ensure recombination occurs throughout the EML, but also have a high photoluminescence quantum yield. The latter is often achieved by mixing the emitter with another wider band gap, higher triplet energy molecule in a host–guest system, which reduces possible aggregation-caused quenching and gives more degrees of freedom in designing the OLED stack by separating charge capture and light emission to different materials in the EML.

For device optimization, a key parameter is the external quantum efficiency (EQE), defined as the ratio of emitted photons to injected charges, thus including both electrical and optical properties. EQE can be expressed as a product of four factors, the first three of which are collectively termed the internal quantum efficiency, IQE:^1^
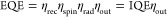
1where η_rec_ is the probability of charge carrier recombination, sometimes also named charge balance, and can be optimized to unity in high-performance devices. η_spin_ represents the proportion of excitons that are theoretically able to decay radiatively and depends on the emission mechanism employed, as outlined in the next paragraphs. η_rad_ is the fraction of excitons that decay radiatively and is usually approximated as the photoluminescence quantum yield (Φ_PL_) of the emitter, which should ideally be unity, and η_out_ is the light out-coupling efficiency, which averages around 20% for isotropic orientation of the transition dipole moments of the emitters within the EML, with the remaining 80% of the light being trapped in waveguide modes by total internal reflection. In short, the EML is involved in most of the individual factors that determine the EQE, so its optimization is of prime importance when working toward an efficient device.

The first OLEDs employed fluorescent emitters, where radiative decay occurred only from the singlet state (see Figure 1a). Due to spin statistics, a quarter of the generated excitons are singlets and three-quarters are triplets. Only the singlet excitons decay radiatively, giving a maximum possible IQE_max_ (internal quantum efficiency, the product of the first three terms in eq 1) of 25%,^3^ which together with an average out-coupling efficiency of 20% gives a maximum external quantum efficiency, EQE_max_, of 5% in the device.

**Figure 1:**
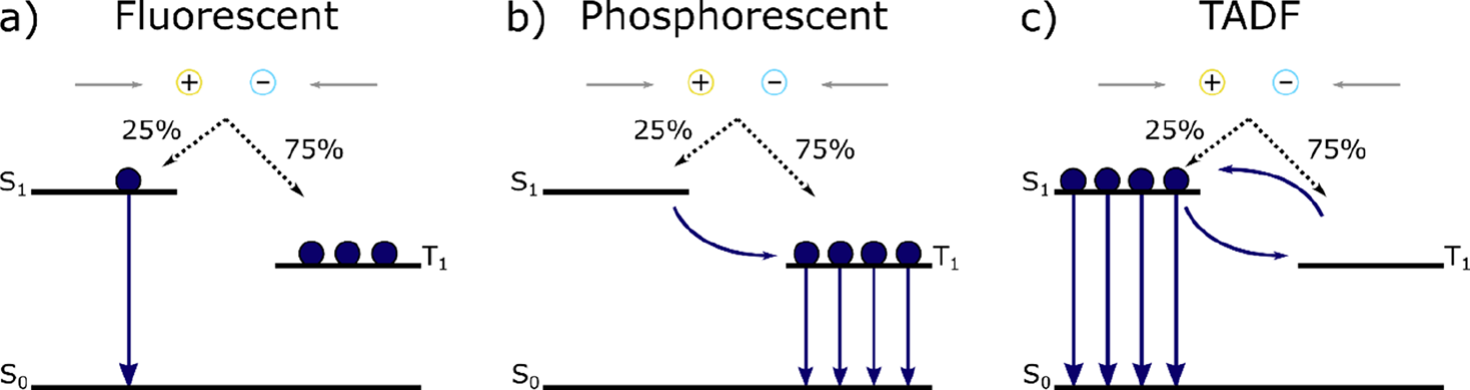
Working principles for fluorescent, phosphorescent and TADF OLEDs.

A considerable improvement came with OLEDs which used phosphorescent emitters.^4^ This mechanism exploits the heavy-atom effect where the presence of the typically used platinoid metals increases spin–orbit coupling and thus accelerates both intersystem crossing (ISC) from singlet to triplet excited states and radiative decay from T_1_ to the ground state. This allows the harvesting of both singlet and triplet excitons and yields light emission by phosphorescence, thus enabling 100% IQE_max_ (Figure 1b). Indeed, the green and red subpixels in commercial OLED displays use iridium(III)-containing emitters, and these devices are stable, having LT50 (the time in which the luminance decreases to 50% of its initial value) values in excess of 10^5^ hours at a luminance of 1000 cd/m^2^.^5^ The major downside of phosphorescent emitters lies in the suboptimal performance of blue analogues to the point where unlike red and green emitters, none are yet commercializable. Industry requires OLEDs that emit at specific CIE (Commission Internationale de l’Éclairage) color coordinates, show high EQE, including at relevant luminance for the application, and have suitably good stability, which necessitates that the blue emitter be bright and be both thermally and photochemically stable.^6^ The specific colors to target are defined according to the standard Rec.2020, outlined by the International Telecommunication Union, which sets the necessary parameters for primary colors in high-resolution displays. For the blue primary color, this corresponds to a monochromatic light source at 467 nm, and coordinates (0.131, 0.046) on the CIE 1931 color space.^7^ While efficient blue emitters have been achieved, the availability of suitable hosts, i.e., with sufficiently high triplet energies and wide bandgaps, is sparse. Another issue arises when considering the sustainability of materials, the design of which often requires the use of scarce heavy metals (platinum, iridium, and palladium) with no established mechanism to readily recycle them after device end-of-life. The abundance of noble metals in Earth’s crust is on the order of 10^–3^ ppm, while, for example, aluminum or silicon, common elements found in electronics, are much more abundant at 10^5^ ppm in the Earth’s crust. This strengthens the need to look for “green” organic semiconductor molecules to preserve the availability of these scarce elements for future generations.^8^

The OLED display industry currently finds a compromise between stability and efficiency by making RGB pixels from red and green phosphorescent emitters and upconverting blue fluorescent emitters. In this case, singlet and triplet energy levels are aligned in a way that allows upconversion via triplet–triplet annihilation (TTA-UC), enabling a theoretical IQE_max_ of 62.5%.^9^ Realistically, achieving high efficiencies with TTA-UC devices is very challenging due to the required involvement of a sensitizer, which is not only responsible for the blue emission but can contribute to singlet quenching from emitter to sensitizer.^10^ This leaves both outstanding materials and device challenges to meet the goal of creating 100% efficient and long-lived blue OLEDs.

The most recent significant advance in emitter design was the introduction of TADF (thermally activated delayed fluorescence) compounds, kickstarting a new class of OLEDs (Figure 1c). Reverse intersystem crossing (RISC) is enabled in compounds that possess a small energy gap between the S_1_ and T_1_ states (Δ*E*_ST_), meaning that in the OLED all triplets could theoretically be harvested for light emission.^11^ However, the rate of RISC is governed not only by the Δ*E*_ST_, but also by both spin–orbit coupling and spin-vibronic interactions. To a first approximation, the RISC rate constant is related to Δ*E*_ST_ by eq 2:
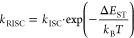
2where *k*_ISC_ and *k*_RISC_ are forward and reverse ISC rate constants, *k*_B_ is the Boltzmann constant and *T* is the temperature.^12^ The equation describing ISC follows from perturbation theory and is given by Fermi’s golden rule:^13^
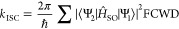
3where Ψ_1_ and Ψ_2_ are the initial and final wave functions, Ĥ _SO_ is the spin–orbit Hamiltonian, and FCWD is the Franck–Condon weighted density of states. The spin–orbit coupling term is the reason why faster ISC and similarly RISC are often observed in molecules with heavy metals, as spin–orbit coupling scales with the fourth power of the atomic number.^14^ This stems from the quantum mechanical nature of the atom, where an electron moving about a nucleus creates a magnetic field, the magnitude of which depends on nuclear charge (atomic number). A heavier atom will cause a stronger magnetic field and thus a larger change in angular momentum, which is the same as spin–orbit coupling (spin angular momentum coupling with orbital angular momentum). Expanding this theory to molecules, spin-flip processes are only possible when spin–orbit coupling is nonzero and this necessitates an accompanied change in orbital type between states to conserve the total angular momentum, referred to as El-Sayed’s rule.^15^

However, when electronic states are close in energy, vibronic states also need to be considered. Their contributions are embedded within eq 3 in the FCWD term, which includes the reorganization energy (a parameter describing changes in geometry between states involved) and vibrational mode energies, both of which are temperature dependent. When vibrational modes between S_0_ and low-energy triplet states are strongly coupled, this spin-vibronic coupling facilitates fast ISC/RISC. The main outcome is that efficient RISC is possible even in organic molecules with small spin–orbit coupling due to the small Δ*E*_ST_ allowing transitions between singlet to triplet states of different character and combining fast RISC with slow nonradiative triplet decay allows for an efficient TADF process.

There are several parameters that need to be optimized in order to achieve the highest performance OLED. An ideal device would have high EQE, low efficiency roll-off (efficiency loss at increased luminance), high stability, a narrowband emission spectrum and appropriate color coordinates to achieve saturated color.^16^ This is particularly challenging for blue OLEDs. Blue OLEDs using phosphorescent emitters where the state-of-the-art materials contain iridium(III) or platinum(II) metal complexes; however, stability is the major issue.^17^ The poor device stability here can be traced back to a combination of thermally accessible nonemissive metal-centered states, too long triplet lifetimes that lead to bimolecular excitonic degradation mechanisms and the inherently weaker metal–ligand bonds that are the primary source of photochemical degradation. One of the inherent difficulties for blue TADF OLEDs to reach the required device metrics also stems mainly from the molecular design of the emitter. Most TADF emitters are based on strongly twisted conformations between electron-donating (D) and -accepting (A) units. This in turn reduces the overlap between the HOMO and LUMO molecular orbitals and produces a sought after small Δ*E*_ST_. While this design increases RISC rates, it reduces the oscillator strength of the S_0_-S_1_ transition, which is reflected in lower Φ_PL_ associated with slower radiative decay rates. Another consequence of the D–A molecular design is that the lowest singlet excited state often exhibits intramolecular charge transfer character, which manifests as a very broad emission band arising from unresolved vibronic bands, giving poor color purity that is a serious stumbling block for display applications. As with phosphorescent emitters, the delayed lifetimes of TADF emitters typically are on the order of microseconds, and so these devices suffer from similar biexcitonic degradation to their PhOLED counterparts.

Simultaneously enhancing all device properties poses a challenge that cannot be solved with simple device structures. One attractive solution is based on the use of sensitizer molecules within the EML that act as efficient exciton harvesters that then transfer the energy to a terminal bright emitter.^18^ The first examples of this strategy employed a phosphorescent sensitizer and a fluorescent terminal emitter, where the phosphor harvested all of the excitons, which were then transferred to the singlet state of another molecule by Förster resonance energy transfer. Thus, exciton harvesting and emission become decoupled on different molecular species, allowing for separate optimization of each process.

The sensitization strategy can be extended from phosphorescent-fluorescent codopant systems to employ TADF emitters as sensitizers, harnessing the strengths of already efficient TADF compounds and addressing their pitfalls.^19^ In a conventional D–A TADF OLED, high efficiency roll-off is a common feature because the exciton lifetime is too long. As the luminance increases, there is a larger concentration of excitons meaning that the probability of bimolecular degradation processes also increases. Specifically, the probability of biexcitonic annihilation (singlet–triplet, singlet–singlet, triplet–triplet, and exciton–polaron) processes increase as the square of exciton concentration, each contributing to a reduction in device efficiency. Further, these annihilation events create excitons that are higher in energy than the bond dissociation energies of the bonds contained within the host and emitter molecules, promoting photochemical dissociation of these bonds, ultimately leading to lower device stability.^20^ Remedying these issues could be achieved by balancing the kinetics of the TADF process as well as energy levels and charge transport properties of the constituent compounds. Optimal TADF rates would reduce the population of long-lived triplet excitons, converting them to emissive singlets. Meanwhile, balanced charge transport would yield a wider recombination zone, decreasing the rates of interfacial recombination events. In this situation TADF-sensitized fluorescence (coined by Adachi et al. as hyperfluorescence)^19^ offers a promising solution, coupling the efficient triplet harvesting of TADF to strongly emissive properties of select fluorescent molecules. It would also contribute to the redistribution of excitons throughout the EML, reducing the probability of bimolecular events and reducing the efficiency roll-off.

Another step forward in the optimization of the device performance is to replace the fluorescent material with a MR-TADF (multiresonant TADF) emitter.^21^ They are typically boron and nitrogen (or oxygen) containing planar polycyclic aromatic hydrocarbon molecules and have short-range charge transfer excited states in such a way that causes globally a large orbital overlap but a relatively small energy difference between lowest singlet and triplet excited states. This means it is possible to have large oscillator strength for transitions to/from the S_1_ state (and consequently large Φ_PL_), and a small Δ*E*_ST_ (permitting the terminal emitter to also contribute to harvesting/recycling of excitons) for the same emitter. Furthermore, the rigid structure of the molecule reduces its vibrational motion, resulting in a very narrow emission spectrum (typically with a full-width at half-maximum of less than 30 nm).^22^ In coupling a MR-TADF terminal emitter (or other high Φ_PL_ narrowband emitting fluorophore) with an efficient assistant dopant sensitizer, it should be possible to achieve a device that reaches all targets outlined for commercial applications. In short, careful consideration about both molecular and device structures is a necessity when designing the next generation of OLEDs.

Blue device degradation is the principal issue to overcome in the development of the next generation OLEDs.^16^ Due to the nature of organic materials in use, the origin of the deterioration is caused by several mechanisms. Historically, the factors impacting device stability have been separated into those that are intrinsic (electrochemical, thermal, interfacial, photochemical, charge balance), and extrinsic (encapsulation, impurities, fabrication environment, substrate, operating conditions).^2^ The intrinsic factors can be summed as those relating to the inner workings of an OLED, while extrinsic factors are associated with device fabrication and operation. Device degradation has also been categorized into catastrophic failure or exponential luminance decay, but this description is not helpful in addressing this issue, as it does not describe the origins of the degradation and thus will not be used here.

Despite being studied since the very advent of the OLEDs, solving intrinsic degradation mechanisms remains difficult owing to the inability to measure directly and dynamically the underlying parameters. As a result, improving device lifetime is generally an iterative process; further, no concrete guidelines exist that would facilitate the pinpointing of the exact cause of the device degradation.^20^ The wide variety of mechanisms, which are also material or device structure dependent, vastly increases the number of variables that determine the end result. In the following paragraphs, we consider different factors leading to OLED degradation, namely, the diffusion of constituent materials, mobile ions, chemical degradation, as well as external degradation factors like variability in device fabrication and encapsulation.

Transport processes play an important role in the overall efficiency and stability of the OLED; hence, it is not far-fetched that polaron diffusion and drift need to be optimized to achieve long device lifetimes.^23^ It has been reported that diffusion of electrically neutral molecules or atoms is possible at driving voltages, leading to defects subsequently acting as exciton quenchers or nonradiative recombination centers.^24^ Migration of ions has also been observed, where the species originate in the electrodes,^25^ and for the commonly used anode ITO (indium tin oxide) it has been shown that indium atoms are effective luminescence quenchers.^26^ Experiments suggest that passivating the ITO surface and thus reducing the indium vacancies is key to minimizing indium migration, with commonly used methods including UV ozone treatment or thin passivation layers (e.g., MoO_3_, LiF, parylene).^23^ Similarly, bilayer cathode materials can react together during the evaporation process and then diffuse into the organic layers.^27^ In the case of Al cathodes, thin interlayers (e.g., LiF, Liq, Mg, ∼1 nm thickness) are often used to reduce their work function by modifying the electronic band structure of Al at the interface. Diffusion of cathode materials can reduce the effective thickness of the adjacent organic layer and diminish the device performance.

An additional sign of degradation within a device is the observed rise of the driving voltage while running it at constant current, which leads to a reduction in luminance efficiency.^28^ The increase in voltage is sometimes found to be reversible by reversing the polarity, and this finding gave rise to a mobile ion model.^29^ This mechanism assumes that there are mobile ion species, originating from degradation of the electrodes or other contaminants, which can redistribute upon application of a voltage and induce an electric field opposite to the external one. Increased ion concentration leads to more nonradiative recombination centers, which is attributed to the decay in luminance. Regions of charge accumulation can also form at the interfaces of transport layers, creating deep traps with the same end result of having nonradiative recombination centers.^30^ Moreover, there is another mechanism relating to the creation of internal electric fields caused by dipole reorientation.^31^ Molecules with a permanent dipole moment are reoriented due to the applied electric field, which then strongly modifies electrical device parameters. The decrease of the device performance could be reversible since it is partially caused by a change in the alignment of molecules, which itself is a reversible process.

Besides charge accumulation, it is necessary to consider how charge balance, a principal parameter affecting the location of exciton formation in the device, impacts both device efficiency and lifetime. While it is well-known that equal amounts of electrons and holes in the emission layer are needed to generate a maximum possible number of excitons and, hence, photons,^20^ charge densities and recombination also need to be taken into account. Excessive quantities of both charge carriers can induce degradation of the emitter molecules as the materials are intrinsically unstable in their ionized state; furthermore, separate chemical degradation pathways could become eminent as these species interact with neutral emitter or host molecules.^32^ A good way to circumvent this issue is to increase the size of the recombination zone, thus reducing the charge density and thus the probability that these degradation pathways can be accessed. Examples of doing this are presented below. The positioning of the recombination zone has also been reported to influence the device lifetime, but the exact optimal location depends on the structure of the OLED, especially the nature of the compounds making up the EML and its adjacent layers.^33^

While all of these mechanisms discussed are important, chemical reactions within the device are also troublesome, and addressing these requires careful consideration. The abundance of charges, excitons, impurities, and Joule heating all contribute to an environment permitting various electrochemical and photochemical reactions in the solid state. The aforementioned ITO decomposition is a good example of such a reaction, as well as well-known cathode reactions with water or oxygen.^34^ Furthermore, gases within the device were shown to form both from moisture adsorbed on the ITO surface, and from decomposition of the organic layers, the latter being a significantly more convoluted problem to solve.^35^ The inherent photochemical instability of organic molecules in their excited state must also be mitigated; hence, careful molecular design is required to minimize the risk of undesired photoredox decomposition reactions occurring, which not only introduces nonradiative pathways, thus diminishing the performance of the device, but also introduces photochemical byproducts that can hinder operation of the OLED.^36^ Particularly for TADF emitters, increasing the bond dissociation energy between donor and acceptor moieties where the weakest bond is normally located would partially address the issue of photochemical instability.^37^

As well as intrinsic degradation factors, there are several external factors that influence the device performance. Since OLED manufacturing via thermal evaporation is a sequential, multistep process, there is plenty of room for variation, whether it is by changing the deposition rate, encapsulants, or cleaning procedures. Each of these parameters are dependent on one another and affect the morphology of the films being deposited, the concentration of defects, and the amount of undesired water and oxygen that become trapped within the device. The number of imperfections in a deposited layer has been shown to depend on the deposition rate;^38^ however, that coupled with the vacuum pressure within the evaporation chamber can also affect how much water vapor and oxygen are incorporated into the device.^39^ The choice of encapsulation material does depend on the type of device such as flexible OLEDs, but the rule of thumb is that the encapsulant must have low water vapor (<10^–6^ g m^–2^ day^–1^) and oxygen (<10^–3^ cm^3^ m^–2^ day^–1^) permeability rates.^40^

One factor that is harder to control is temperature, an increase of which not only changes device characteristics and emitter photophysics but can accelerate degradation^41^ or induce total device failure.^42^ For some applications OLEDs need to be capable of operating in up to 90 °C,^43^ which poses design challenges in term of the stability of these devices. An increase in thermal energy accelerates chemical reactions within the device, and with higher temperature, a change in layer morphology becomes probable as a cause of catastrophic failure.^44^ The morphology change when a glassy material becomes rubbery happens at the glass transition temperature *T*_g_, which should be as high as possible to increase the stability of the OLED. Thus, not only does the inherent thermal stability of the materials need to be considered but so too does the processing of the films and the fabrication protocols of the devices.

The long list of possible degradation mechanisms raises the question of which ones are of greater importance and thus should be addressed first. A reasonable first approach would be to tackle those that also impact other device characteristics such as EQE_max_ or efficiency roll-off. This stems from the assumption that optimizing charge balance, exciton distribution and fabrication procedures are necessary to manufacture a high-performing device, and optimizing these will likely kill two birds with one stone. It is important to realize that typically solutions to improve device lifetime come at a trade-off in efficiency; for example, changing a transport material to one that is more chemically stable, but having lower charge mobility.

For any new scientific breakthrough to be commercialized, standardized testing procedures need to be established and testing must be robust and reproducible. Such testing also benefits research in general, giving clear targets to aim for and instructions on how to report achievements so that they are consistent within the field. Despite this, and unlike the field of solar cells where national testing facilities exist and testing protocols are widely adopted,^45^ the few standards concerning OLEDs that exist at the moment only relate to OLED tiles and panels.^46,47^ It is clear that standardization is needed for an orderly improvement of small-scale devices, and there are good examples of how to do it from the field of solar energy, especially since the issue of stability when employing organic materials is shared.^40^

With the increasing use of renewable energy technology, photovoltaics (PVs) have become an important contributor to the global electricity production, and thus research into PV technology remains a fast growing area of study.^48^ One facet of PV research is to develop a class of organic PVs, which would extend the capabilities of solar energy harvesting beyond what is achievable with conventional silicon cells. A consensus has been reached that described methodologies, known as ISOS (International Summit on OPV Stability) protocols, for testing and reporting PV degradation,^49^ and a later follow-up commentary extended these protocols to thin-film devices.^50^ The key message that can be taken from these papers is that it is paramount to not only report lifetime values but also consider that the rate of device degradation is not consistent due to the variety of mechanisms at play. Thus, reporting only device lifetime values does not provide sufficient information to guide improvements in device stability performance.

Another notable point relates to accelerated lifetime studies and their limits. To avoid months-long measurements for stable devices, it is common to measure devices at higher stress conditions and then apply a Coulombic degradation scaling law,^51^ which relates the initial luminance to the measured lifetime (eq 4):
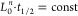
4where *L*_0_ is initial luminance, *t*_1/2_ is device half-life, and *n* is the scaling factor, which is typically between 1.7 and 1.8, but should be measured by fitting a plot of lifetime dependence on initial luminance.^52−54^ However, the different profiles of the degradation curves mean that this relation may not always be true, particularly when various decay mechanisms act on different time scales. To obtain accurate and meaningful device stability data, it is therefore necessary to not extrapolate over large magnitudes of time from a short initial measurement.^55^

To improve the accuracy and value of device lifetime measurements beyond accelerated aging studies, statistical models can be used, where devices are tested under harsh conditions (i.e., higher current, temperature) and a statistical distribution of lifetimes can be obtained, which would account for the variability among the individual units tested.^56^ The rate of degradation under different stress conditions can point to the main causes of device degradation.^55^

Industry performance metrics for the production of OLEDs are stringent, requiring the achievement of concomitantly suitable color purity, luminance, and lifetime. With respect to device lifetime, a LT95 (the time in which the luminance decreases to 95% of its initial value) of at least 5000 h and 1000 cd/m^2^ luminance is expected of the OLED to be competitive with modern day technologies such as HD televisions and mobile phone displays.^16^ However, because of a lack of clearly defined testing standards, reported device lifetime values range from LT50 (the time to 50% of the initial luminance) to LT97, making it nearly impossible to compare device stability data directly. In this Review, lifetimes will be cited renormalized according to the Coulombic degradation scaling law^43^ with an acceleration factor of 1.8 to an initial luminance of 1000 cd/m^2^ unless stated otherwise, with conclusions made focusing on the qualitative device stability. When comparing efficiencies, it would be preferable to report EQE at a useful brightness of 1000 cd/m^2^, but due to many authors not including this information, only EQE_max_ values are reported here. For the field to advance to real applications, it is necessary for researchers to consider and accurately report efficiencies at a practical luminance, as that is the parameter that needs to be better than that of devices currently in use. A full list of reported values is found in the Supporting Information (Tables S1–S5).

The first OLED displayed a LT50 of 100 h, starting from an initial luminance of only 50 cd/m^2^.^57^ Since then, red and green phosphorescent OLEDs have made remarkable improvements and are now integral in every commercialized OLED display product, with LT50 values in excess of 10^5^ hours (initial luminance of 1000 cd/m^2^). However, the blue OLEDs still use fluorescent-based TTA-UC emitters, which reach just over 10^4^ hours, not only showing less than ideal stability, but much poorer external quantum efficiencies.^58^ Furthermore, this mismatch of lifetimes between different colored pixels leads to a serious problem of differential aging as the color balance changes. One potential solution would be to replace the blue OLEDs with ones employing TADF emitters, so long as these devices exhibit the required improved EQE, efficiency roll-off, and stability.

The importance of developing both efficient and long-lasting devices becomes evident when surveying literature performance metrics for blue OLEDs, where a large array of compounds were investigated as emitters (Figure 2) and hosts (Figure 3). While some still assert that blue OLEDs based on fluorescent molecules are the only ones capable of reaching reasonable lifetimes,^59^ recent progress to improve their efficiency to match that of red and green devices has hit a ceiling in term of their EQE_max_. There have been consistent efforts at producing stable OLEDs that operate by only harvesting singlet excitons for emission, but despite significant improvements in device lifetime, the EQE_max_ was at best half of what could be achieved with phosphorescent or TADF emitters. Other approaches to developing efficient and stable devices include the use of well-known MR-TADF emitter **DABNA-1** and its derivative **t-DABNA** (Figure 2a). The role of the host was assigned to **α-ADN** (Figure 3a), which has a high singlet energy, needed for exciton transfer to the blue MR-TADF terminal emitter, and a low-lying triplet level, suitable for triplet exciton quenching. The devices with **DABNA-1** and **t-DABNA** showed deep blue emission at CIE coordinates of (0.126, 0.098) and (0.135, 0.072), respectively; however, the EQE_max_ for the devices with 5 wt % emitter concentration in the host was only 5.5 and 7.8%, while the LT95 values were 2.7 and 10.0 h (when renormalized to an initial luminance of 1000 cd/m^2^), respectively.^59^ In another study, a donor–acceptor material based on pyrene **Py(5,9)BDPA** (Figure 2b) was used with α,β-ADN (Figure 3a) as the host to develop a TTA-UC OLED. It produced a blue color at (0.132, 0.27), and an extrapolated LT95 of 534 h; however, the EQE_max_ was just over 4%.^60^ Another report disclosed a device with a LT95 of just under 500 h (LT50 of 8000 h) and an EQE_max_ of 11.9% with a different pyrene-based compound as the emitter (Figure 2b) and **CzPA** (Figure 3b) as the host in a TTA-UC system.^52^ In terms of color, while the peak of the EL spectrum was at a reasonably blue 466 nm, it also included a strong, red-shifted component, likely arising from an aggregate state, and consequently the device emitted at CIE coordinates of (0.14, 0.16). In summary, despite the efforts to increase the EQE in devices relying on TTA-UC, it is still very far from the already efficient phosphorescent and TADF OLEDs.

**Figure 2:**
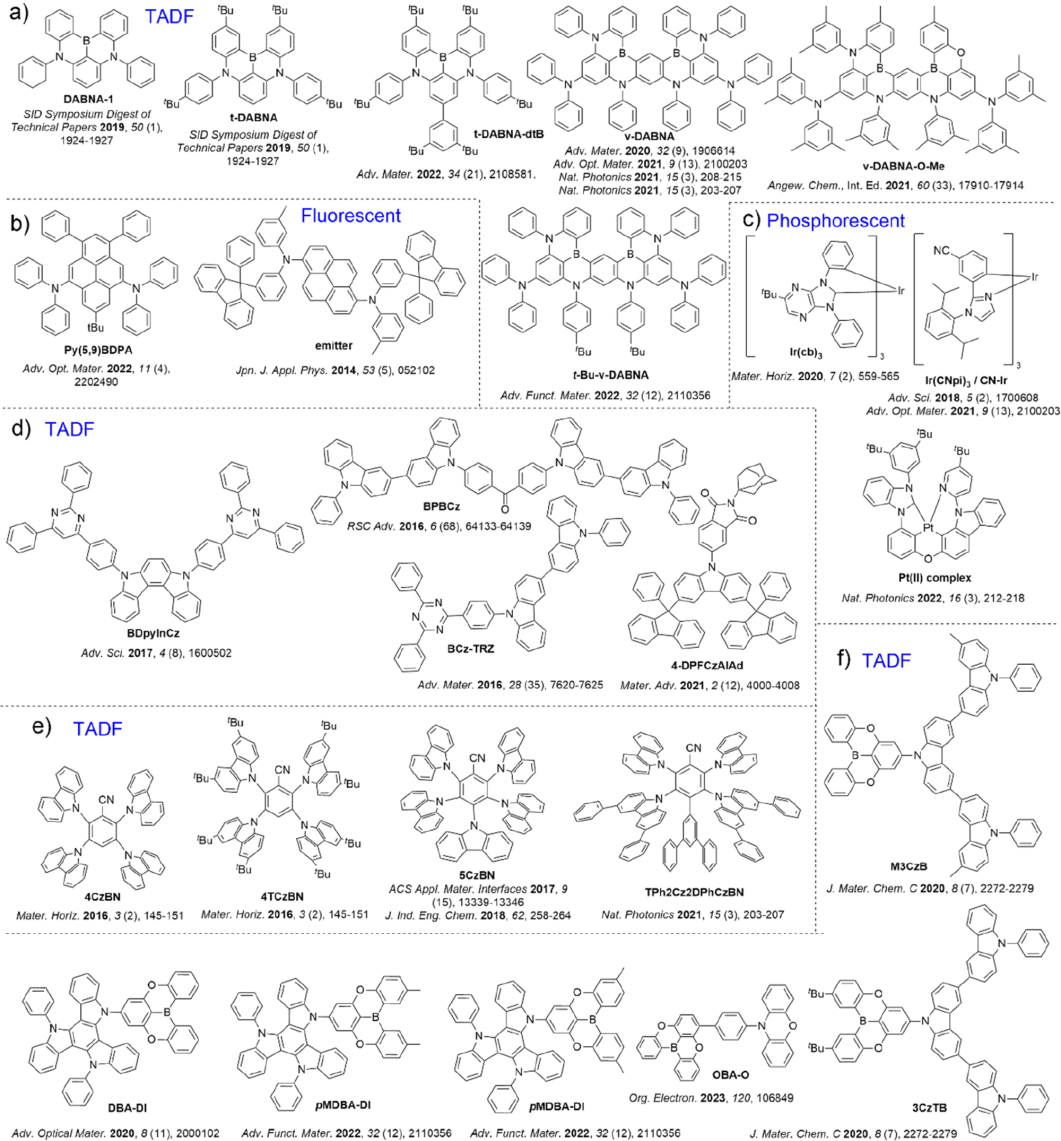
Structures of emitter materials.

**Figure 3:**
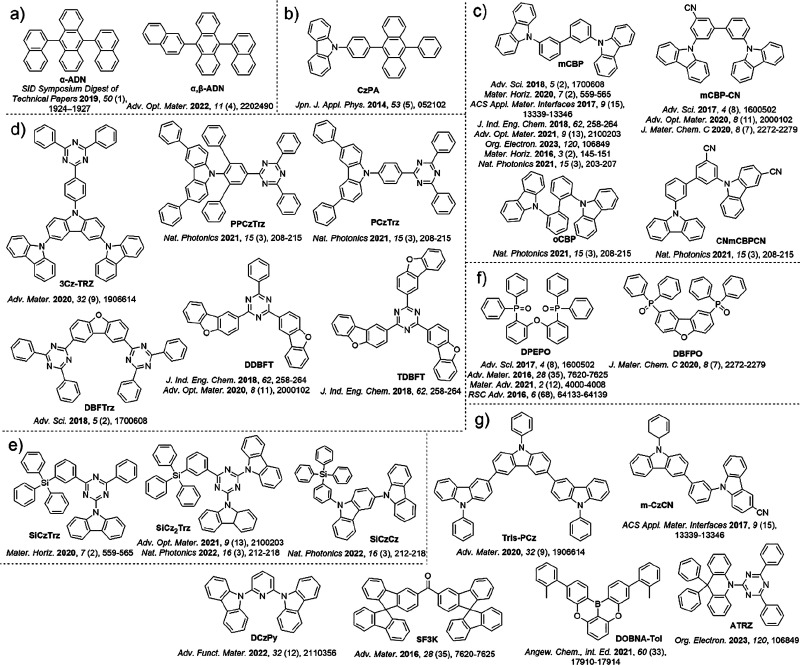
Structures of host materials.

Since the nature of fluorescent OLEDs means that their IQE has an upper limit of just 25%, phosphorescent OLEDs are a logical evolution to higher performance devices. In most of the OLEDs, the performance of the emitter depends on the intimate interactions with the surrounding host matrix. The host must be a material that possesses a larger HOMO–LUMO gap and a higher triplet energy than the emitter to prevent a loss channel of triplets moving from the emitter to host via Dexter energy transfer.^61^ The high triplet energies of the host needed for blue OLEDs (close to 3 eV) come close to the bond dissociation energies of many of the bonds in organic semiconductor materials. Worse, annihilation events such as TTA, as well as a similar triplet-polaron annihilation (TPA) mechanism, are driven by the triplet density and thus are more prominent when the device is run at a high current density. A proven tactic to alleviate the nonradiative and degradation pathways accessible due to both TTA and TPA is to exchange the single host for a mixed-host or an exciplex host system.^54^ A mixed-host works by using two hosts in the EML, with one selected for electron transport and the other optimized for hole transport, thus expanding the recombination zone and reducing exciton density.^62^ Additionally, positive and negative polarons become trapped on the respective host, allowing recombination to occur directly on the emitter, bypassing the exciton transfer step. Exciplex type hosts are likewise composed of electron transport and hole transport compounds that are held together by intermolecular interaction. Such hosts operate in a manner similar to that for the mixed-host system by separating the charge transport channels and reducing exciton density. However, excitons can be generated on the exciplex host and subsequently move to the emitter via energy transfer. Owing to the relatively weakly electronically coupled donor and acceptor molecules within the exciplex, these materials frequently exhibit TADF, and thus the host can act to synergistically harvest excitons prior to energy transfer to the emitter guest within the EML. The downside of exciplexes is that their triplet energy needs to be above the band gap of the emitter, and the energies of the constituent components need to be even larger to account for the exciton formation energy, which is an extremely difficult task for blue.^54^

Nevertheless, careful designs of the host–guest system have been reported that lead to higher efficiencies of OLEDs than in fluorescent OLEDs without any dramatic losses of stability. One innovative approach employed the use of an electroplex host^63^ – a system working very similarly to that of the exciplex, differing by the fact that an electroplex is formed only from injected charges, arising from its constituent compounds having opposite electron donating and accepting character. Because of this, it has a smaller exciton binding energy, making access to host–guest systems with higher energy emission easier compared with the use of an exciplex host. An electroplex host for blue phosphorescent OLEDs was developed by combining carbazole-type and triazine-type hosts **mCBP** (Figure 3c) and **DBFTrz** (Figure 3d). Both compounds have triplet energies of >2.8 eV, making them good candidates for blue emitters. Devices with the emitter **Ir(CNpi)**_**3**_ (Figure 2c) doped into the electroplex host were made to evaluate stability and compared against devices with single-host systems. OLEDs with **mCBP**:**DBFTrz** achieved an EQE_max_ of 18% with color coordinates of (0.16, 0.29), showing a tangible improvement over devices with single hosts (EQE_max_ values of 12.8% and 12.7% for devices using **mCBP** and **DBFTrz** hosts, respectively). Furthermore, the device with the electroplex system exhibited higher stability, having an LT50 of 11.2 h (compared to 2.8 h for the device using **mCBP** and 1.3 h for the device using **DBFTrz**). Another implementation of an electroplex host for blue OLEDs used **mCBP** with **SiCzTrz** (Figure 3e) as the electron transport host, and the emitter **Ir(cb)**_**3**_. The device showed blue emission at (0.12, 0.13) with an EQE_max_ of 27.6% and an LT50 of 170 h, which was interpreted as due to the high polaron stability of both host materials making up the electroplex.^54^ An even greater improvement in stability was demonstrated by developing both a new Pt-based emitter **PtON-TBBI** (Figure 2c) and two silyl-based host materials, which formed an exciplex host.^64^ OLEDs with **PtON-TBBI** and using the **SiCzCz**:**SiTrzCz**_**2**_ (Figure 3e) exciplex host showed blue emission at (0.141, 0.197) with EQE_max_ of 25.4%, a remarkably low efficiency roll-off with EQE at 1000 cd/m^2^ of 23.4%, and an impressive LT95 of 150 h (reported LT70 was 1113 h). The resulting stability was attributed to an increased intrinsic stability of the emitter bearing a tetracoordinate ligand, and improved polaron stability of the silylated host compounds compared to those without the silyl groups.

We next consider the lifetimes of TADF-based OLEDs. In this case, one of the principal issues remains identifying an appropriate and stable host. A relatively simple way to increase device lifetime is by swapping out the widely used but unstable phosphine oxide-based hosts, such as **DPEPO** (Figure 3f). **DPEPO** is an electron-transport type host, and has a very shallow LUMO level, leading to both electrons and holes to be transported at least in part directly through the emitter, putting electrical stress on it and losing one advantage of the host–guest system.^65^ In a study aiming to prove this, the host **mCBP-CN** (Figure 3c) was used in an OLED with a high concentration of the blue TADF emitter **BDpyInCz** (Figure 2d), and the device performance cross-compared with reference devices made with **DPEPO** as the host.^65^ The **mCBP-CN**: 20% emitter devices showed an EQE_max_ of 13.6% at color coordinates of (0.173, 0.266) and had an LT80 of 6 h. The devices with **DPEPO** showed a slightly higher EQE_max_ of 15% and red-shifted emission at (0.211, 0.359), but had significantly shorter LT80 of 8 min, suggesting that the choice of host is paramount for stable OLEDs. The **mCBP-CN** host likewise provides more balanced charge transport compared to the commonly used host, **mCBP**, which has unbalanced charge transport.^66^ In another study, a novel host, **m-CzCN** (Figure 3g) was used in conjunction with the blue TADF emitter **5CzBN** (Figure 2e), and the device was cross-compared with similar devices but with **mCBP** acting as the host. The OLEDs with **m-CzCN**:**5CzBN** achieved an EQE_max_ of 15% and had LT70 of 11 h, whereas the **mCBP**-based devices showed only a 9.3% EQE_max_ and had an LT70 of 6 h (emission was around 480 nm for both devices). The 2-fold increase in device lifetime was explained to be the result of the more stable **m-CzCN** host material coupled with improved electron transport within the EML.

A different strategy to improve upon **mCBP** as a host is to add electron transporting type cohosts such as **DDBFT** and **TDBFT** (Figure 3d).^67^ Devices with **5CzBN** as the emitter using the mixed **DDBFT**:**mCBP** host showed EQE_max_ of 10.2% at CIE coordinates of (0.18, 0.35) and an LT50 of 41.6 h. For comparison, devices with only **mCBP** as the host showed an EQE_max_ of 9.0% at almost the same color of (0.18, 0.34), but showed a much shorter LT50 of 17.7 h. Similarly, the device using only **DDBFT** as the host achieved an EQE_max_ of only 6.6% at (0.19, 0.37) and an LT50 of 18.1 h; the use of the other novel host **TDBFT** produced devices of comparable performance using a mixed host system, yet when used as a single host, the devices showed an EQE_max_ of 6.4% at color coordinates (0.19, 0.39) and exhibited the shortest LT50 of 11.5 h. This highlights the need to not only consider the intrinsic host stability but also balance the charge transport within the EML. A different strategy targeted increasing the thermal stability of the host by creating a high *T*_g_ material.^68^ The host **ATRZ** (Figure 3g) not only showed a higher *T*_g_ compared to **mCBP** (115 °C versus 92 °C), but also had a higher triplet level at 3.07 eV (**mCBP** has the T_1_ at 2.81 eV).^69^ Devices with the sky-blue MR-TADF emitter **OBA-O** (Figure 2f) showed an EQE_max_ of 10.2% at color coordinates of (0.19, 0.32) and had an LT50 of 1.2 h using this novel host. Meanwhile devices with **mCBP** as the host showed slightly lower EQE_max_ of 8.5% and a red-shifted electroluminescence at (0.19, 0.36) and a shorter LT50 of 30 min. While improvements were achieved due to the improved charge transport in the EML and the higher *T*_g_ of **ATRZ**, further adjustments are needed to both reach longer lifetimes and address the high efficiency roll-off present in all devices described in this report.

Further investigations into suppressing emitter degradation led to the design of novel TADF compounds that are relatively more stable to both electrochemical and photo-oxidation.^70^ The OLED with the sky-blue emitter **BCz-TRZ** (Figure 2d) and using **DPEPO** as the host showed an EQE_max_ of 20.5% and an LT50 of 9 h at λ_EL_ of around 490 nm. The devices were modified by swapping out the host for the more stable **SF3K** (Figure 3g), which led to an increase of the lifetime (LT50 of 37 h) at the cost of greatly reducing the EQE_max_ to 10.4%. In a different study, the bicarbazole moiety was investigated as a strong donor in the TADF emitter, aiming to improve stability by having a higher bond dissociation energy, while maintaining a small Δ*E*_ST_.^71^ The resulting sky-blue OLED [CIE coordinates of (0.21, 0.34)] with **BPBCz** (Figure 2d) and using **DPEPO** host had an LT50 value of around 20 min, and while it showed an EQE_max_ of 23.3%, there was significant efficiency roll-off (EQE_1000_ of 10.3%). This suggests that there was a significant effect from bimolecular annihilation events, reducing both stability and EQE at high brightness, which would need to be addressed before a conclusion can be formulated concerning the emitter stability. A similar approach, but more focused on increasing efficiency, was used in a report of two new blue TADF emitters, **3CzTB** and **M3CzB** (Figure 2f) containing tercarbazole donors coupled to boron-based acceptors.^72^ OLEDs with these two emitters were separately optimized for their EQE_max_ and lifetimes. Devices using **DBFPO** (Figure 3f) as the host material reached an EQE_max_ of 29.1% (**3CzTB**) and 30.7% (**M3CzB**) at color coordinates of (0.14, 0.19) and (0.14, 0.26); however, their lifetimes were not measured. The structure that was used for lifetime testing instead employed **mCBP-CN** as the host, with devices achieving significantly lower efficiencies and bluer emission than their **DBFPO** counterparts: EQE_max_ of 7.6% at (0.14, 0.10) with an LT50 of 12 h for the device with **3CzTB**, and EQE_max_ of 14.4% at (0.13, 0.19) with an LT50 of 16 h for the device with **M3CzB**. While no degradation mechanisms were investigated, this study is a good example of how optimizing an OLED for stability often comes at the cost of efficiency.

With the goal of devices that exhibit simultaneously high efficiency and stability, reducing chemical degradation in emitters with high Φ_PL_ values becomes a primary goal. In one study, a strategy of sterically shielding the emissive core of a blue emitter with *tert*-butyl units was explored.^53^ Blue TADF OLEDs with **4TCzBN** (Figure 2e) using **mCBP** as the host were compared against those with **4CzBN** (Figure 2e), the analogous emitter without the *tert*-butyl substituents. The devices with **4TCzBN** reached an EQE_max_ of 16.2% at CIE coordinates of (0.16, 0.22) and had an LT50 of 48 h, which is an improvement over the device with **4CzBN** (EQE_max_ of 10.6% at similar color coordinates of (0.17, 0.20) and with an LT50 of 18 h), suggesting that the increased intermolecular separation was responsible for the reduced exciton-polaron annihilation, thus elongating device lifetime. Similarly, in another report even larger bulky substituents were shown to be more beneficial compared to *tert*-butyl groups.^73^ An emitter **4-DPFCzAIAd** containing adamantyl substituents (Figure 2d) was used in the EML together with **DPEPO** as the host, producing a device with an impressive EQE_max_ of 28.2% and sky-blue emission at (0.20, 0.36), together with an LT50 of 51 h. Despite this, the high efficiency roll-off (EQE_1000_ of only 6.4%) still needs to be addressed.

From all of these aforementioned reports, it is clear that charge transport management in the EML is of prime importance, and acceptable results are unlikely with a simple host–guest system. Here, sensitization strategies are worth examining, starting with TADF-sensitized emission (commonly termed hyperfluorescence, HF). The generally accepted definition of a HF-OLED requires an EML to consist of a suitably high triplet energy host, a TADF-type assistant dopant that acts as a sensitizer and a terminal emitter that emits from its singlet excited state;^74^ however, the term has come to be used by some much more expansively to encompass any sensitized exciton harvesting system that leads to fluorescence.^75^ Using the definition involving TADF sensitization, the assistant dopant **TPh2Cz2DPhCzBN** (Figure 2e) has been developed to be compatible with the MR-TADF pure-blue emitter **ν-DABNA**.^76^ Energy transfer in a film consisting of **mCBP** as the host was found to be very efficient (Figure 2a), suggesting that an EML of the composition **ν-DABNA:TPh2Cz2DPhCzBN:mCBP** could be used to produce a stable and efficient OLED. For reference, devices with a simple host:guest configuration were made with both **TPh2Cz2DPhCzBN** and **ν-DABNA** in **mCBP**. The conventional OLED with **ν-DABNA** showed a poor EQE_max_ of 3.7% with emission at CIE coordinates of (0.12, 0.11) and a short lifetime (LT95 < 1 h), while the device with **TPh2Cz2DPhCzBN** exhibited an EQE_max_ of 22% at CIE coordinates of (0.19, 0.40) and showing an improved LT95 of 29 h. The HF devices yielded an EQE_max_ of 27%, relatively lower efficiency roll-off (EQE_1000_ of 20%) and emission at CIE coordinates of (0.15, 0.20). The modest LT95 of 11 h was rationalized in terms of the increased charge trapping on the terminal emitter. A different report showcased two TADF materials *p***MDBA-DI** and *m***MDBA-DI** (Figure 2f) acting as assistant dopants in HF OLEDs in conjunction with the terminal emitter *t***-Bu-ν-DABNA** (Figure 2a).^77^ HF OLEDs were optimized for stability using **DCzPy** (Figure 3g) as the host and cross-compared to their assistant dopant only TADF OLED counterparts. The devices with *p***MDBA-DI** showed an EQE_max_ of 23.0% at CIE coordinates of (0.16, 0.33) and possessed a LT50 of 332 h, while the devices with *m***MDBA-DI** achieved a similar EQE_max_ of 21.3% at CIE coordinates of (0.15, 0.26) but were much less stable, reflected in an order of magnitude shorter LT50 of 35 h. The HF devices showed an expected blue-shift in the emission due to the use of the narrowband terminal emitter [CIE coordinates of (0.13, 0.13) for the device with *p***MDBA-DI** and (0.12, 0.16) for the device with *m***MDBA-DI**], together with higher EQE_max_ of 26.6 and 28.1%, and longer LT50 of 440 and 133 h, respectively, for the devices employing *p***MDBA-DI** and *m***MDBA-DI**. These examples serve as strong evidence that segregating exciton harvesting and emission on different photoactive compounds is a useful strategy for increasing device stability without a trade-off in efficiency.

A less conventional example of an attempt to address charge transport in the EML involved the use of an exciplex-based host system where one of the host materials constituting the exciplex exhibited TADF behavior (Figure 4a).^78^ The authors hypothesized that by substituting the regular fluorescent acceptor of the exciplex with one that is TADF, there would be a reduction in the accumulation of long-lived triplet states on the acceptor and consequently an increase in the stability of the exciplex system due to reduced TTA rates. A stable exciplex system with **Tris-PCz** (Figure 3g) and 3Cz-TRZ (Figure 3d) was developed, which exhibited broad green emission. The blue OLEDs with 1 wt % **ν-DABNA** doped into equal ratios of **Tris-PCz** and **3Cz-PCz** showed sky-blue emission at (0.29, 0.36) with an EQE_max_ of 19%; the device showed low efficiency roll-off with an EQE_1000_ above 18% and had an LT50 value of over 450 h. While it was demonstrated that the use of the exciplex was compatible with the fabrication of blue devices, the emission spectrum has a green component resulting from the exciplex host system, thus reducing color purity. The goal of reducing triplet population on the emitter has inspired other modifications to the EML structure, such as using an intermediate phosphor with energy levels between the host and the TADF emitters.^79^ In one study, the EML consisted of a mixed-host **mCBP**:**SiCz**_**2**_**Trz**, a phosphor **CN-Ir** (Figure 2c) and the terminal MR-TADF emitter **ν-DABNA** (Figure 4b). Device performance was compared between those with and without the phosphor codopant, demonstrating that the OLED with **CN-Ir** achieved a higher EQE_max_ of 27.3% at color coordinates (0.13, 0.16) and had a longer LT50 of 121 h compared to the device with only **ν-DABNA** [EQE_max_ of 19.5% at CIE coordinates of (0.12, 0.12) and LT50 of 26 h].^79^

**Figure 4:**
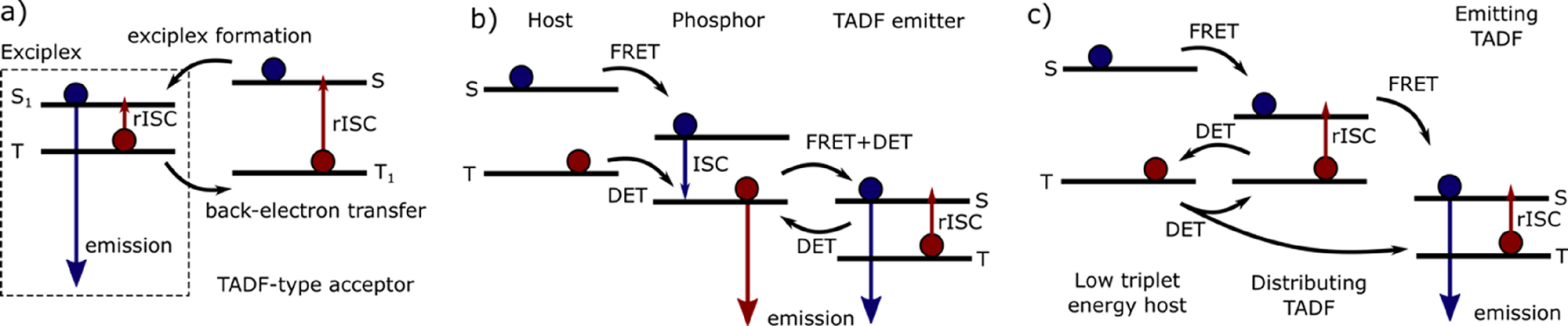
Principal schemes for exciton harvesting mechanisms including (a) an exciplex with a TADF-type acceptor, (b) an intermediate phosphor, (c) a distributing TADF compound. Singlet and triplet states without indices correspond to a range of excited states.

Another strategy employed for deep-blue OLEDs used two TADF materials to manage triplet population, one of which was responsible for exciton harvesting and the other of which was responsible for emission (Figure 4c).^80^ Here a lower triplet energy host can be used, with its quenched triplets recycled by the distributing TADF assistant dopant. Exciton harvesting TADF materials **PPCzTrz** and **PCzTrz** (Figure 3d) were compared in an EML also consisting of the mixed **oCBP**:**CNmCBPCN** (Figure 3c) host and emitter **ν-DABNA**. The OLEDs used with **PPCzTrz** and **PCzTrz** achieved EQE_max_ of 33.0 and 33.5%, both showing blue emission at (0.13, 0.20) and (0.12, 0.18) and having LT50 of 151 and 113 h, all, respectively. Comparing these two devices to one with only **ν-DABNA** and the same mixed-host, the EQE_max_ was not improved (33.2%), but the LT50 value of the latter conventional device was only 41 h, with the differences in stability rationalized by the careful management of the exciton population by the assistant TADF dopants.

A deeper assessment of the devices with MR-TADF emitters reveals that there is still much room for improvement. For example, devices with **ν-DABNA-O-Me** (Figure 2a) doped in **DOBNA-Tol** (Figure 3g) host showed deep-blue emission at (0.13, 0.10) color coordinates and a high EQE_max_ of 29.5% coupled with low efficiency roll-off (EQE_1000_ of 26.9%); however, the LT50 was only 5 h.^81^ Another study used **t-DABNA-dtB** (Figure 2a) as the emitter where OLEDs using a mixed host **mCBP**:**mCBP-CN** showed extremely high efficiency roll-off and short lifetimes (EQE_max_ of 25.4%, EQE_1000_ of 3%, LT50 of 2.8 h), so the authors pivoted to using the MR-TADF compound as a TTA-UC emitter.^82^ This resulted in a device with reduced EQE_max_ of 11.4%, but significantly improved roll-off (EQE_1000_ of 10.9%) and a much improved LT95 value of 208 h.

Taking previous degradation studies into account, the most stable blue TADF OLED to date was achieved using a boron-based emitter **DBA-DI** (Figure 2f) having high bond dissociation energies and electrochemical stability and using a mixed host system **mCBP-CN**:**DDBFT** having high triplet energies.^83^ The OLED showed a high EQE_max_ of 26.4%, low efficiency roll-off (EQE_1000_ of 26.3%) and an LT50 value of 540 h (LT95 of 17.3 h); the devices emitted in the sky-blue at color coordinates (0.17, 0.40), so some optimization is needed to achieve the primary blue color standard. The device performance was mostly attributed to a wide recombination zone, coupled with the stability of the emitter.

Summarizing the large body of work linked to improving the lifetimes of the OLEDs, it is geared toward producing bluer and more stable efficient devices. This can be visualized by a graphical summary of reported OLED LT95 values versus the dominant wavelength emitted by the devices (Figure 5). OLEDs in the red color region have attracted less attention as state-of-the-art devices largely have acceptably long device lifetimes, with the few exceptions being OLEDs for specific applications (e.g., needing a specific deep-red color point,^84^ large area,^85^ very high brightness).^86^ Meanwhile, there are plenty of studies devoted to improving the lifetimes of green OLEDs, largely focusing on employing very efficient TADF emitters and concurrently aiming to reduce the efficiency roll-off that is usually present in TADF-based devices. Methods for improving device performance include creative EML designs that employ components like sensitizers, electroplex hosts, TADF hosts, etc., (named TADF+ in Figure 5). Similarly, there has been much research focused on blue OLEDs both in academia and in industry, with Universal Display Corporation aiming to deploy phosphorescent blue emitters into the commercial market in 2024^87^ and Kyulux planning to launch hyperfluorescent blue devices the same year.^88^ Despite this, the lifetimes of blue devices are still about an order of magnitude shorter than those for green devices, leaving a vast area for improvement.

**Figure 5:**
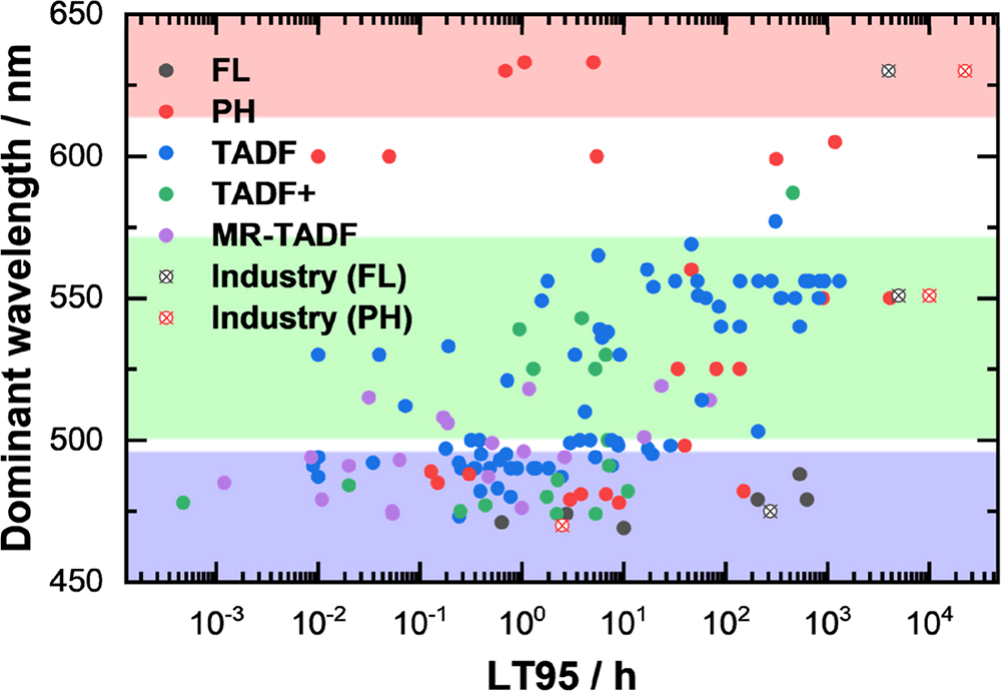
Reported OLED LT95 values, sampled focusing on blue devices, and reported lifetimes of OLEDs used in industry.^5,21,52−54,59,60,64−68,70−73,76−84,89−111^

Addressing the challenge of improving the device lifetimes for blue OLEDs has been a major focus of research. Studies on device degradation paved the way to understanding possible degradation pathways. Limiting the diffusion of materials within the device, whether atoms, small molecules, or mobile ion species, is required to maintain the integrity of the structure of the device and avoid luminance quenching. Furthermore, choosing appropriate, inherently stable materials with optimal charge transport is needed to yield the benefits of a wide recombination zone. Reducing both charge and exciton concentrations is paramount to suppressing photochemical degradation reactions, as is appropriate control of Joule heating. A careful consideration of all steps of the device fabrication from the choice of structure to its encapsulation is also required to prevent external factors negatively affecting device performance.

Learning from the aforementioned work, making an efficient and stable blue device should involve starting with photochemically stable, bright TADF-type emitters. To help balance exciton kinetics, the use of assistant dopants is likely required, such that triplet harvesting and light emission processes are segregated to different molecules. The host should be a mixed-host or exciplex system with high triplet levels and wide bandgaps for all components, allowing for balanced electron and hole transport and avoiding charge transport through the dopant, thus reducing the electrical stress on emitter molecules. Layers adjacent to the EML should have optimal energy levels for charge injection into the EML, as well as high triplet levels to minimize the chances of triplet quenching. Electrodes should not only provide efficient charge injection but also have low resistance to reduce Joule heating and be sufficiently stable to minimize diffusion of their constituents into organic layers. All in all, it takes a considerable amount of methodical planning to devise a device structure that would be resistant to degradation, and further research is needed to continue improvement in this area to address industry requirements.

## Supporting Information Available

Methodology, tables of reported OLED lifetime values (PDF)
