# Supplementary material for: The Blue Problem: OLED Stability and Degradation Mechanisms
Source: J Phys Chem Lett. 2024 Jan 23;15(4):1034–47. doi: 10.1021/acs.jpclett.3c03317 (PMC10839906; doi:10.1021/acs.jpclett.3c03317)
Supplement: Supplementary file 1 — jz3c03317_si_001.pdf [file jz3c03317_si_001.pdf]

Supporting information

The Blue Problem: OLED Stability and Degradation Mechanisms

Eglė Tankelevičiūtė[a,b], Ifor D. W. Samuel[b] and Eli Zysman-Colman*[a]*

[a]Organic Semiconductor Centre, EaStCHEM School of Chemistry, University of St Andrews, St Andrews, UK, KY16 9ST. E-mail: eli.zysman-colman@st-andrews.ac.uk

[b]Organic Semiconductor Centre, School of Physics & Astronomy, University of St Andrews, St Andrews, UK, KY16 9SS. E-mail: idws@st-andrews.ac.uk

Contents

Methodology S1
Reported OLED lifetime values S2
References S12

Methodology

OLED type, CIE coordinates, device lifetime values and initial luminance for device lifetime measurements were gathered from published reports where available. Dominant wavelength was computed from CIE coordinates using a calculator from Luminus (website: https://luminus-cie1931-demo.anvil.app) and was used instead of peak electroluminescence wavelength to better represent how the color would look to the human eye.

LT95 and LT50 values were extracted from the reported device lifetime graphs where available using WebPlotDigitizer: Version 4.6 (author: Ankit Rohatgi, website: https://automeris.io/WebPlotDigitizer). LT95 values were then renormalized using the acceleration factor of 1.8

in the Coulombic degradation law,[1] and rounded up to 3 significant figures. The expected error for these values is in the region of 5% of the stated value.

Reported OLED lifetime values

Table **S1**. Fluorescent OLED characteristics.

Reported					Extracted			Normalized	
Emitter	CIE[a] / (x, y)	LT[b]	Time[c] / h	L_0[d] / cd m^{-2}	λ[e] / nm	LT95[f] / h	LT50[g] / h	LT95[f] / h	Ref
t-DABNA-dtB	0.12, 0.13	95	208	1000	478	208	-	208	2
t-DABNA	0.14, 0.07	95	181.6	200	469	181.6	-	10.0	3
DABNA-1	0.13, 0.10	95	49.5	200	474	49.5	-	2.73	3
PyCN	0.14, 0.09	95	11.5	200	471	11.5	-	0.63	3
Py(5,9)BDPA	0.13, 0.27	95	534	1000	488	534	-	534	4
Pyrene-based	0.14, 0.16	50	800	5000	479	36.0	-	634	5
-	0.67, 0.33	50	160000	1000	630	-	160000	4000	6
-	0.31, 0.63	50	200000	1000	551	-	200000	5000	6
-	0.14, 0.12	50	11000	1000	475	-	11000	275	6

[a] Color coordinates in the CIE 1931 color space, [b] Relative luminance (in %) at which device lifetime is measured, [c] Reported lifetime, [d] Initial luminance, [e] Dominant wavelength, [f] Time in which luminance decreases to 95%, [g] Time in which luminance decreases to 50%.

Table **S2**. Phosphorescent OLED characteristics

Reported						Extracted		Normalized	
Emitter	CIE[a] / (x, y)	LT[b]	Time[c] / h	L_0[d] / cd m^{-2}	λ[e] / nm	LT95[f] / h	LT50[g] / h	LT95[f] / h	Ref
$Ir(cb)_3$	0.14, 0.19	50	5900	100	481	240	5900	3.80	7
$Ir(cb)_3$	0.14, 0.19	50	8460	100	481	422	8460	6.69	7
$Ir(cb)_3$	0.12, 0.13	50	10700	100	478	570	10700	9.03	7
$Ir(ppy)_3$	-	80	1000	2000	525	9.79	499	34.1	8
$Ir(ppy)_3$	-	80	383	2000	525	23.4	-	81.6	8
$Ir(ppy)_3$	-	80	95	2000	525	39.7	-	138	8
$Ir(mphmq)_2(tmd)$	0.64, 0.35	90	2243	1000	605	1187	-	1190	9
$Ir(MDQ)_2(acac)$	0.62, 0.38	90	1102	1000	599	315	-	315	9
$Ir(mphmq)_2(tmd)$	-	90	0.2	1000	600	0.01	0.31	0.01	9
$Ir(mphmq)_2(tmd)$	-	90	74	1000	600	5.43	-	5.43	9
$Ir(ppy)_2(acac)$ + $Ir(MDQ)_2(acac)$	-	90	1.5	1000	600	0.05	11.5	0.05	9
$(DPQ)_2Ir(dpm)$	0.63, 0.30	70	250	170	630	16.7	-	0.69	10
$(DPQ)_2Ir(dpm)$	0.68, 0.29	70	375	170	633	25.9	-	1.06	10
$(DPQ)_2Ir(dpm)$	0.70, 0.29	70	890	170	633	123	-	5.06	10
$(mdppy)_2Iracac$	0.37, 0.60	90	11	5000	560	2.55	-	46.2	11
$Ir(ppy)_3$	-	90	407	5000	550	228	-	4120	12
$Ir(ppy)_3$	-	90	140	5000	550	49.8	-	903	12
Pt(II) complex	0.13, 0.15	95	3	1000	479	3	-	3.00	13
Pt(II) complex	0.22, 0.39	95	40	1000	498	40	-	40.0	13
Pt(II) complex	0.14, 0.20	95	150	1000	482	150	-	150	13

$Ir(CNpi)_3$	0.15, 0.24	50	9.7	500	485	0.52	9.7	0.15	[14]
$Ir(CNpi)_3$	0.16, 0.30	50	4.4	500	489	0.45	4.4	0.13	[14]
$Ir(CNpi)_3$	0.16, 0.29	50	38.9	500	488	1.06	38.9	0.30	[14]
-	0.64, 0.36	50	900000	1000	630	-	900000	22500	[6]
-	0.31, 0.63	50	400000	1000	551	-	400000	10000	[6]
-	-	50	100	1000	460	-	100	2.50	[6]

[a] Color coordinates in the CIE 1931 color space, [b] Relative luminance (in %) at which device lifetime is measured, [c] Reported lifetime, [d] Initial luminance, [e] Dominant wavelength, [f] Time in which luminance decreases to 95%, [g] Time in which luminance decreases to 50%.

Table **S3**. TADF OLED characteristics.

Reported					Extracted			Normalized	
Emitter	CIE[a] / (x, y)	LT[b]	Time[c] / h	L_0[d] / cd m^{-2}	λ[e] / nm	LT95[f] / h	LT50[g] / h	LT95[f] / h	Ref
BCz-TRZ	0.18, 0.34	50	32	500	490	0.88	32	0.25	15
3Cz-TRZ	0.18, 0.32	50	2.8	296	491	0.08	2.8	0.01	15
BCz-TRZ	-	50	63	500	500	1.34	63	0.38	15
BCz-TRZ	0.18, 0.34	50	80	500	490	1.22	80	0.35	15
BCz-TRZ	0.18, 0.34	50	45	500	490	1.7	45	0.49	15
BCz-TRZ	0.18, 0.34	50	130	500	490	1.19	130	0.34	15
5CzCN	-	70	6	1000	490	0.78	-	0.78	16
5CzCN	-	70	6	1000	490	0.9	-	0.90	16
5CzCN	-	70	11	1000	490	1.3	-	1.30	16
DMAC-TRZ	0.23, 0.48	50	0.066	3000	512	0.01	0.07	0.07	17
BPBCz	0.21, 0.34	50	0.1	2000	492	0.01	0.13	0.03	17
TrzBCz	0.21, 0.36	50	0.133	1000	494	0.01	0.11	0.01	17
M3CzB	0.13, 0.19	50	81	400	482	2.04	81	0.39	18
3CzTB	0.14, 0.10	50	60.5	400	473	1.26	60.5	0.24	18
5CzCN	0.18, 0.34	50	17.7	1000	492	0.24	17.7	0.24	19
5CzCN	0.19, 0.37	50	18.1	1000	495	0.4	18.1	0.40	19
5CzCN	0.19, 0.39	50	11.5	1000	497	0.18	11.5	0.18	19
5CzCN	0.18, 0.35	50	41.6	1000	493	0.61	41.6	0.61	19
5CzCN	0.19, 0.37	50	41.7	1000	495	0.7	41.7	0.70	19
4CzBN	0.17, 0.20	50	62	500	480	2.7	62	0.78	20

4TCzBN	0.16, 0.22	50	167	500	483	2.02	167	0.58	[20]
5CzBN	0.22, 0.40	50	176	500	499	10.4	176	3.00	[20]
5TCzBN	0.21, 0.41	50	770	500	500	16.4	770	4.72	[20]
5CzCN	0.17, 0.27	80	100	500	487	8.55	-	2.46	[21]
4-CzAIAd	0.20, 0.32	50	16	500	490	6.4	16	1.84	[22]
4-TBCzAIAd	0.26, 0.48	50	34	500	521	2.51	34	0.72	[22]
4-DPFCzAIAd	0.20, 0.36	50	178	500	494	18.4	178	5.28	[22]
DBA-DI	0.15, 0.33	50	48	1000	491	7.67	48	7.67	[23]
DBA-DI	0.16, 0.39	50	329	1000	495	19.1	329	19.1	[23]
DBA-DI	0.17, 0.41	50	348	1000	498	8.85	348	8.85	[23]
DBA-DI	0.17, 0.40	50	540	1000	497	17.3	540	17.3	[23]
4CzIPN	0.22, 0.51	90	30	2000	514	16.83	-	58.6	[24]
4CzIPN	-	50	353	5986	540	3.6	353	90.2	[25]
4CzIPN	-	50	418	6537	540	4.71	418	138	[25]
4CzIPN	-	50	524	6510	540	18.45	524	538	[25]
5TCzBN	-	50	220	1290	500	2.36	220	3.73	[25]
5TCzBN	-	50	223	1259	500	0.21	223	0.32	[25]
5TCzBN	-	50	300	1261	500	5.06	300	7.68	[25]
4CzIPN	-	80	250	1000	530	9.16	-	9.16	[26]
4CzIPN	-	80	90	1000	530	3.32	-	3.32	[26]
4CzIPN	0.34, 0.59	90	485	1000	556	211	-	211	[27]
4CzIPN	0.34, 0.58	90	630	1000	556	283	-	283	[27]
4CzIPN	0.34, 0.58	90	1115	1000	556	654	-	654	[27]
4CzIPN	0.34, 0.59	90	290	1000	556	140	-	140	[27]
4CzIPN	0.34, 0.59	90	140	1000	556	52.9	-	52.9	[27]

4CzIPN	0.34, 0.59	90	30	1000	556	1.79	-	1.79	[27]
4CzIPN	0.34, 0.58	90	1130	1000	556	671	-	671	[27]
4CzIPN	0.34, 0.58	90	1380	1000	556	844	-	844	[27]
4CzIPN	0.34, 0.58	90	1045	1000	556	623	-	623	[27]
4CzIPN	0.34, 0.58	90	1200	1000	556	604	-	604	[27]
4CzIPN	0.34, 0.58	95	935	1000	556	935	-	935	[27]
4CzIPN	0.34, 0.58	95	1315	1000	556	1315	-	1320	[27]
5CzCN	0.19, 0.41	97	3	1000	500	8.7	-	8.70	[28]
3Cz2DPhCzBN	0.21, 0.44	97	110	1000	500	209	-	209	[28]
5TCzBN	-	50	475	500	510	14.5	475	4.16	[12]
DMAC-BP	0.26, 0.55	50	3.5	1000	533	0.2	2.48	3.44	[29]
DMAC-BP	-	50	0.34	1000	530	0.0	0.34	0.72	[29]
DMAC-BP	-	50	0.35	1000	530	0.01	0.35	0.01	[29]
BTrzICz	0.30, 0.57	95	86	1000	547	86	-	86.0	[30]
BTrzBCz	0.31, 0.58	95	64	1000	550	64	-	64.0	[30]
4CzIPN	0.34, 0.60	95	32	1000	556	32	-	32.0	[30]
ICBNTrz1	0.27, 0.56	90	24.2	1000	538	7.04	-	7.04	[31]
ICBNTrz2	0.31, 0.55	90	4	1000	549	1.57	-	1.57	[31]
ICBNTrz3	0.32, 0.55	90	139.5	1000	551	54.0	-	54.0	[31]
ICBNTrz4	0.25, 0.51	90	13.3	1000	536	6.17	-	6.17	[31]
4CzIPN	0.33, 0.60	90	58.5	1000	554	19.6	-	19.6	[31]
CzAcSF	0.17, 0.28	40	0.2	1000	487	0.0	0.15	0.01	[32]
6,7-DCNQx-DICz	0.49, 0.50	90	931	1000	577	309	-	309	[33]
5,8-DCNQx-DICz	0.55, 0.44	90	1184	1000	587	459	-	459	[33]
4CzIPN-Me	0.37, 0.58	50	1470	1000	560	16.9	1470	16.9	[34]

4CzIPN	-	50	636	5951	550	33.2	636	824	[35]
4CzIPN	-	50	626	5460	550	16.8	626	356	[35]
4CzIPN	-	50	639	5340	550	17.1	639	348	[35]
4CzIPN	-	50	497	6248	550	17.9	497	483	[35]
1	0.16, 0.31	50	2354	100	490	86.8	2354	1.38	[36]
2	0.28, 0.54	50	12733	100	539	370	12733	5.86	[36]
3	0.40, 0.56	50	9495	100	565	357	9495	5.66	[36]
TPh2Cz2DPhCzBN	0.19, 0.40	95	29	1000	498	29	-	29	[37]

[a] Color coordinates in the CIE 1931 color space, [b] Relative luminance (in %) at which device lifetime is measured, [c] Reported lifetime, [d] Initial luminance, [e] Dominant wavelength, [f] Time in which luminance decreases to 95%, [g] Time in which luminance decreases to 50%.

Table **S4**. TADF+ OLED characteristics. TADF+ OLEDs employ components like sensitizers, exciplex hosts, TADF hosts etc.

Reported					Extracted			Normalized	
EML	CIE[a] / (x, y)	LT[b]	Time[c] / h	L_0[d] / cd m^{-2}	λ[e] / nm	LT95[f] / h	LT50[g] / h	LT95[f] / h	Ref
mCBP:$SiCz_2Trz$:**ν-DABNA**	0.12, 0.12	50	26	1000	477	0.44	26	0.44	38
mCBP:$SiCz_2Trz$:CN-Ir:**ν-DABNA**	0.13, 0.16	50	121	1000	480	1.76	121	1.76	38
oCBP:CNmCBPCN:PPCZTrz:**ν-DABNA**	0.12, 0.09	50	117	1000	474	5.32	117	5.32	39
oCBP:CNmCBPCN:PCzTrz:**ν-DABNA**	0.12, 0.09	50	97	1000	474	2.21	97	2.21	39
mCBP:$Ir(cb)_3$:**t-DABNA**	0.13, 0.11	50	293	200	475	4.50	293	0.25	40
DPEPO:DMAC-DPS:**t-DABNA**	0.13, 0.15	50	30	100	478	0.03	0.5	0.00	41
CzAcSF:**TBPe**	0.15, 0.23	40	0.9	1000	484	0.02	0.39	0.02	32
mCBP:4CzIPN-Me:**TBRb**	0.43, 0.54	50	3775	1000	569	46.3	3775	46.3	34
TrisPCz:3Cz-TRZ	0.26, 0.53	50	337	830	530	9.28	337	6.64	42
TrisPCz:BCz-TRZ	0.26, 0.50	50	292	1050	525	4.82	292	5.26	42
TrisPCz:Cz-TRZ	0.29, 0.55	50	123	920	543	4.50	123	3.87	42
TrisPCz:SF3-TRZ	0.25, 0.52	50	93	780	525	2.04	93	1.30	42
TrisPCz:T2T	0.28, 0.54	50	66	1030	539	0.90	66	0.95	42
**ν-DABNA**:TrisPcz:3Cz-TRZ	0.29, 0.36	50	320	1260	500	4.58	320	6.94	42

mCBP:**ν-DABNA**: TPh2Cz2DPhCzBN	0.15, 0.20	95	11	1000	482	11	-	11	[37]
DCzPy:*p*MDBA-DI: ***t*-Bu-ν-DABNA**	0.16, 0.33	50	440	1000	491	7.20	133	7.20	[43]
DCzPy:*m*MDBA-DI: ***t*-Bu-ν-DABNA**	0.15, 0.26	50	133	1000	486	2.25	440	2.25	[43]

[a] Color coordinates in the CIE 1931 color space, [b] Relative luminance (in %) at which device lifetime is measured, [c] Reported lifetime, [d] Initial luminance, [e] Dominant wavelength, [f] Time in which luminance decreases to 95%, [g] Time in which luminance decreases to 50%.

Table **S5**. MR-TADF OLED characteristics.

Reported					Extracted			Normalized	
Emitter	CIE[a] / (x, y)	LT[b]	Time[c] / h	L_0[d] / cd m^{-2}	λ[e] / nm	LT95[f] / h	LT50[g] / h	LT95[f] / h	Ref
ν-DABNA-O-Me	0.13, 0.10	50	314	100	474	3.4	314	0.05	44
TDBA–DI	0.14, 0.27	50	55.2	1000	487	0.47	55.2	0.47	45
TDBA–DI	0.17, 0.39	50	62.8	1000	496	1.05	62.8	1.05	45
TDBA–DI	0.17, 0.36	50	102.9	1000	494	2.62	103	2.62	45
t-DABNA	-	50	17	200	475	0.97	17	0.05	40
t-DABNA	-	50	30	100	479	0.69	31	0.01	41
TW-BN	0.14, 0.36	50	10.4	500	493	0.22	10.4	0.06	46
TPh-BN	0.10, 0.46	50	36.5	500	499	1.78	36.5	0.51	46
pCz-BN	0.13, 0.54	50	27.7	500	506	0.65	27.7	0.19	46
mCz-BN	0.15, 0.55	50	18.6	500	508	0.59	18.6	0.17	46
2F-BN	0.16, 0.60	90	45.8	2000	514	20.09	-	70.0	24
3F-BN	0.20, 0.58	90	15.5	2000	519	6.76	-	23.5	24
4F-BN	0.12, 0.48	90	10.4	2000	501	4.55	-	15.8	24
OBA-O	0.19, 0.32	50	2.4	800	491	0.03	2.4	0.02	47
OBA-O	0.19, 0.36	50	0.6	973	494	0.009	0.6	0.01	47
OBA-O	0.17, 0.25	50	0.08	1107	485	0.001	0.08	0.00	47
BN-DMAC	0.19, 0.59	80	82	500	518	4.13	-	1.19	48
BN-DPAC	0.16, 0.61	80	8	500	515	0.11	-	0.03	48
ν-DABNA	0.12, 0.11	95	1	1000	476	1	-	1	37

[a] Color coordinates in the CIE 1931 color space, [b] Relative luminance (in %) at which device lifetime is measured, [c] Reported lifetime, [d] Initial luminance, [e] Dominant wavelength, [f] Time in which luminance decreases to 95%, [g] Time in which luminance decreases to 50%.

References

(1) Zhang, W.; Wu, Z.; Liang, S.; Jiao, B.; Zhang, X.; Wang, D.; Hou, X.; Chen, Z.; Gong, Q. Study on scalable Coulombic degradation for estimating the lifetime of organic light-emitting devices. *J. Phys. D: Appl. Phys.* **2011**, *44* (15), 155103.

(2) Park, J.; Kim, K. J.; Lim, J.; Kim, T.; Lee, J. Y. High efficiency of over 25% and long device lifetime of over 500 h at 1000 nit in blue fluorescent organic light-emitting diodes. *Adv. Mater.* **2022**, *34* (21), 2108581.

(3) Lee, K. H.; Lee, J. Y.; Oh, H. Y. P-184: Boron derivatives as deep blue fluorescent materials for high efficiency and long lifetime. In *SID Symposium Digest of Technical Papers*, 2019; Wiley Online Library: Vol. 50, pp 1924-1927.

(4) Xie, F.; Yang, X.; Jin, P.; Wang, X. T.; Ran, H.; Zhang, H.; Sun, H.; Su, S. J.; Hu, J. Y. Achieving simultaneously ultrahigh brightness, extremely low efficiency roll-off and ultralong lifetime of blue fluorescent OLEDs by using donor-acceptor-type 5,9-diarylamine functionalized pyrenes. *Adv. Opt. Mater.* **2022**, *11* (4), 2202490.

(5) Suzuki, T.; Nonaka, Y.; Watabe, T.; Nakashima, H.; Seo, S.; Shitagaki, S.; Yamazaki, S. Highly efficient long-life blue fluorescent organic light-emitting diode exhibiting triplet–triplet annihilation effects enhanced by a novel hole-transporting material. *Jpn. J. Appl. Phys.* **2014**, *53* (5), 052102.

(6) Sudheendran Swayamprabha, S.; Dubey, D. K.; Yadav, R. A. K.; Nagar, M. R.; Sharma, A.; Tung, F. C.; Jou, J. H. Approaches for long lifetime organic light emitting diodes. *Adv. Sci.* **2021**, *8* (1), 2002254.

(7) Jung, M.; Lee, K. H.; Lee, J. Y.; Kim, T. A bipolar host based high triplet energy electroplex for an over 10000 h lifetime in pure blue phosphorescent organic light-emitting diodes. *Mater. Horiz.* **2020**, *7* (2), 559-565.

(8) Hirai, H.; Nakajima, K.; Nakatsuka, S.; Shiren, K.; Ni, J.; Nomura, S.; Ikuta, T.; Hatakeyama, T. One-Step borylation of 1, 3-diaryloxybenzenes towards efficient materials for organic light-emitting diodes. *Angew. Chem., Int. Ed.* **2015**, *54* (46), 13581-13585.

(9) Lee, J.-H.; Shin, H.; Kim, J.-M.; Kim, K.-H.; Kim, J.-J. Exciplex-forming co-host-based red phosphorescent organic light-emitting diodes with long operational stability and high efficiency. *ACS Appl. Mater. Interfaces* **2017**, *9* (4), 3277-3281.

(10) Nagai, Y.; Sasabe, H.; Takahashi, J.; Onuma, N.; Ito, T.; Ohisa, S.; Kido, J. Highly efficient, deep-red organic light-emitting devices using energy transfer from exciplexes. *J. Mater. Chem. C* **2017**, *5* (3), 527-530.

(11) Guo, K.; Wang, H.; Wang, Z.; Si, C.; Peng, C.; Chen, G.; Zhang, J.; Wang, G.; Wei, B. Stable green phosphorescence organic light-emitting diodes with low efficiency roll-off using a novel bipolar thermally activated delayed fluorescence material as host. *Chem. Sci.* **2017**, *8* (2), 1259-1268.

(12) Zhang, D.; Wei, P.; Zhang, D.; Duan, L. Sterically shielded electron transporting material with nearly 100% internal quantum efficiency and long lifetime for thermally activated delayed fluorescent and phosphorescent OLEDs. *ACS Appl. Mater. Interfaces* **2017**, *9* (22), 19040-19047.

(13) Sun, J.; Ahn, H.; Kang, S.; Ko, S.-B.; Song, D.; Um, H. A.; Kim, S.; Lee, Y.; Jeon, P.; Hwang, S.-H. Exceptionally stable blue phosphorescent organic light-emitting diodes. *Nat. Photonics* **2022**, *16* (3), 212-218.

(14) Song, W.; Lee, J. Y.; Cho, Y. J.; Yu, H.; Aziz, H.; Lee, K. M. Electroplex as a new concept of universal host for improved efficiency and lifetime in red, yellow, green, and blue phosphorescent organic light-emitting diodes. *Adv. Sci.* **2018**, *5* (2), 1700608.
(15) Cui, L. S.; Deng, Y. L.; Tsang, D. P. K.; Jiang, Z. Q.; Zhang, Q.; Liao, L. S.; Adachi, C. Controlling synergistic oxidation processes for efficient and stable blue thermally activated delayed fluorescence devices. *Adv. Mater.* **2016**, *28* (35), 7620-7625.
(16) Byeon, S. Y.; Kim, J. H.; Lee, J. Y. CN-modified host materials for improved efficiency and lifetime in blue phosphorescent and thermally activated delayed fluorescent organic light-emitting diodes. *ACS Appl. Mater. Interfaces* **2017**, *9* (15), 13339-13346.
(17) Kim, H. M.; Choi, J. M.; Lee, J. Y. Blue thermally activated delayed fluorescent emitters having a bicarbazole donor moiety. *RSC Adv.* **2016**, *6* (68), 64133-64139.
(18) Karthik, D.; Ahn, D. H.; Ryu, J. H.; Lee, H.; Maeng, J. H.; Lee, J. Y.; Kwon, J. H. Highly efficient blue thermally activated delayed fluorescence organic light emitting diodes based on tercarbazole donor and boron acceptor dyads. *J. Mater. Chem. C* **2020**, *8* (7), 2272-2279.
(19) Kang, Y. J.; Han, S. H.; Lee, J. Y. Lifetime enhancement of blue thermally activated delayed fluorescent devices by separated carrier channels using dibenzofuran-triazine type hosts. *J. Ind. Eng. Chem.* **2018**, *62*, 258-264.
(20) Zhang, D.; Cai, M.; Zhang, Y.; Zhang, D.; Duan, L. Sterically shielded blue thermally activated delayed fluorescence emitters with improved efficiency and stability. *Mater. Horiz.* **2016**, *3* (2), 145-151.
(21) Cho, Y. J.; Jeon, S. K.; Lee, J. Y. Molecular engineering of high efficiency and long lifetime blue thermally activated delayed fluorescent emitters for vacuum and solution processed organic light-emitting diodes. *Adv. Opt. Mater.* **2016**, *4* (5), 688-693.
(22) Feng, Q.; Zheng, X.; Wang, H.; Zhang, H.; Qian, Y.; Tan, K.; Cao, H.; Xie, L.; Huang, W. A 9-fluorenyl substitution strategy for aromatic-imide-based TADF emitters towards efficient and stable sky blue OLEDs with nearly 30% external quantum efficiency. *Mater. Adv.* **2021**, *2* (12), 4000-4008.
(23) Ahn, D. H.; Maeng, J. H.; Lee, H.; Yoo, H.; Lampande, R.; Lee, J. Y.; Kwon, J. H. Rigid oxygen-bridged boron-based blue thermally activated delayed fluorescence emitter for organic light-emitting diode: approach towards satisfying high efficiency and long lifetime together. *Adv. Opt. Mater.* **2020**, *8* (11), 2000102.
(24) Zhang, Y.; Zhang, D.; Wei, J.; Liu, Z.; Lu, Y.; Duan, L. Multi-resonance induced thermally activated delayed fluorophores for narrowband green OLEDs. *Angew. Chem., Int. Ed.* **2019**, *58* (47), 16912-16917.
(25) Kamata, T.; Sasabe, H.; Ito, N.; Sukegawa, Y.; Arai, A.; Chiba, T.; Yokoyama, D.; Kido, J. Simultaneous realization of high-efficiency, low-drive voltage, and long lifetime TADF OLEDs by multifunctional hole-transporters. *J. Mater. Chem. C* **2020**, *8* (21), 7200-7210.
(26) Im, Y.; Song, W.; Lee, J. Y. Effect of the molecular structure of the host materials on the lifetime of green thermally activated delayed fluorescent organic light-emitting diodes. *J. Mater. Chem. C* **2015**, *3* (31), 8061-8065.
(27) Tsang, D. P.-K.; Matsushima, T.; Adachi, C. Operational stability enhancement in organic light-emitting diodes with ultrathin Liq interlayers. *Sci. Rep.* **2016**, *6* (1), 22463.
(28) Noda, H.; Nakanotani, H.; Adachi, C. Excited state engineering for efficient reverse intersystem crossing. *Sci. Adv.* **2018**, *4* (6), eaao6910.
(29) Zhang, Q.; Tsang, D.; Kuwabara, H.; Hatae, Y.; Li, B.; Takahashi, T.; Lee, S. Y.; Yasuda, T.; Adachi, C. Nearly 100% internal quantum efficiency in undoped electroluminescent devices employing pure organic emitters. *Adv. Mater.* **2015**, *27* (12), 2096-2100.
(30) Lee, H. L.; Lee, K. H.; Lee, J. Y.; Lee, H. J. Molecular design opening two emission pathways for high efficiency and long lifetime of thermally activated delayed fluorescent organic light-emitting diodes. *J. Mater. Chem. C* **2021**, *9* (23), 7328-7335.

(31) Lee, H. J.; Lee, H. L.; Han, S. H.; Lee, J. Y. Novel secondary acceptor based molecular design for superb lifetime in thermally activated delayed fluorescent organic light-emitting diodes through high bond energy and fast up-conversion. *Chem. Eng. J.* **2022**, *427*, 130988.
(32) Song, W.; Lee, I.; Lee, J. Y. Host engineering for high quantum efficiency blue and white fluorescent organic light-emitting diodes. *Adv. Mater.* **2015**, *27* (29), 4358-4363.
(33) Kothavale, S.; Chung, W. J.; Lee, J. Y. High efficiency and long lifetime orange-red thermally activated delayed fluorescent organic light emitting diodes by donor and acceptor engineering. *J. Mater. Chem. C* **2021**, *9* (2), 528-536.
(34) Furukawa, T.; Nakanotani, H.; Inoue, M.; Adachi, C. Dual enhancement of electroluminescence efficiency and operational stability by rapid upconversion of triplet excitons in OLEDs. *Sci. Rep.* **2015**, *5* (1), 1-8.
(35) Abe, S.; Sasabe, H.; Nakamura, T.; Matsuya, M.; Saito, Y.; Hanayama, T.; Araki, S.; Kumada, K.; Kido, J. Effect of substitution position of dibenzofuran-terminated robust hole-transporters on physical properties and TADF OLED performances. *Mol. Syst. Des. Eng.* **2023**, *8*, 388.
(36) Li, P.; Chan, H.; Lai, S. L.; Ng, M.; Chan, M. Y.; Yam, V. W. W. Four-Coordinate boron emitters with tridentate chelating ligand for efficient and stable thermally activated delayed fluorescence organic light-emitting devices. *Angew. Chem., Int. Ed.* **2019**, *58* (27), 9088-9094.
(37) Chan, C.-Y.; Tanaka, M.; Lee, Y.-T.; Wong, Y.-W.; Nakanotani, H.; Hatakeyama, T.; Adachi, C. Stable pure-blue hyperfluorescence organic light-emitting diodes with high-efficiency and narrow emission. *Nat. Photonics* **2021**, *15* (3), 203-207.
(38) Chung, W. J.; Lee, K. H.; Jung, M.; Lee, K. M.; Park, H. C.; Eum, M. S.; Lee, J. Y. Over 30 000 h device lifetime in deep blue organic light-emitting diodes with y color coordinate of 0.086 and current efficiency of 37.0 cd A^{-1}. *Adv. Opt. Mater.* **2021**, *9* (13), 2100203.
(39) Jeon, S. O.; Lee, K. H.; Kim, J. S.; Ihn, S.-G.; Chung, Y. S.; Kim, J. W.; Lee, H.; Kim, S.; Choi, H.; Lee, J. Y. High-efficiency, long-lifetime deep-blue organic light-emitting diodes. *Nat. Photonics* **2021**, *15* (3), 208-215.
(40) Lee, K. H.; Lee, J. Y. Phosphor sensitized thermally activated delayed fluorescence organic light-emitting diodes with ideal deep blue device performances. *J. Mater. Chem. C* **2019**, *7* (28), 8562-8568.
(41) Han, S. H.; Jeong, J. H.; Yoo, J. W.; Lee, J. Y. Ideal blue thermally activated delayed fluorescence emission assisted by a thermally activated delayed fluorescence assistant dopant through a fast reverse intersystem crossing mediated cascade energy transfer process. *J. Mater. Chem. C* **2019**, *7* (10), 3082-3089.
(42) Nguyen, T. B.; Nakanotani, H.; Hatakeyama, T.; Adachi, C. The role of reverse intersystem crossing using a TADF-type acceptor molecule on the device stability of exciplex-based organic light-emitting diodes. *Adv. Mater.* **2020**, *32* (9), 1906614.
(43) Naveen, K. R.; Lee, H.; Braveenth, R.; Karthik, D.; Yang, K. J.; Hwang, S. J.; Kwon, J. H. Achieving high efficiency and pure blue color in hyperfluorescence organic light emitting diodes using organo-boron based emitters. *Adv. Funct. Mater.* **2022**, *32* (12), 2110356.
(44) Tanaka, H.; Oda, S.; Ricci, G.; Gotoh, H.; Tabata, K.; Kawasumi, R.; Beljonne, D.; Olivier, Y.; Hatakeyama, T. Hypsochromic shift of multiple-resonance-induced thermally activated delayed fluorescence by oxygen atom incorporation. *Angew. Chem., Int. Ed.* **2021**, *60* (33), 17910-17914.
(45) Ahn, D. H.; Kim, S. W.; Lee, H.; Ko, I. J.; Karthik, D.; Lee, J. Y.; Kwon, J. H. Highly efficient blue thermally activated delayed fluorescence emitters based on symmetrical and rigid oxygen-bridged boron acceptors. *Nat. Photonics* **2019**, *13* (8), 540-546.
(46) Liu, F.; Cheng, Z.; Wan, L.; Feng, Z.; Liu, H.; Jin, H.; Gao, L.; Lu, P.; Yang, W. Highly efficient multi-resonance thermally activated delayed fluorescence material with a narrow full width at half-maximum of 0.14 eV. *Small* **2022**, *18* (4), 2106462.

(47) Kreiza, G.; Banevičius, D.; Juršėnas, S.; Rodella, F.; Strohriegl, P.; Kazlauskas, K. Ambipolar hosts for blue TADF OLEDs: Assessment of the device performance and lifetime. *Org. Electron.* **2023**, *120*, 106849.
(48) Jiang, P.; Zhan, L.; Cao, X.; Lv, X.; Gong, S.; Chen, Z.; Zhou, C.; Huang, Z.; Ni, F.; Zou, Y. Simple acridan-based multi-resonance structures enable highly efficient narrowband green TADF electroluminescence. *Adv. Opt. Mater.* **2021**, *9* (21), 2100825.
